# Placenta-Derived MicroRNAs in the Pathophysiology of Human Pregnancy

**DOI:** 10.3389/fcell.2021.646326

**Published:** 2021-03-11

**Authors:** Peng Xu, Yeling Ma, Hongyu Wu, Yan-Ling Wang

**Affiliations:** ^1^School of Life Science, Shanxi University, Taiyuan, China; ^2^State Key Laboratory of Stem Cell and Reproductive Biology, Institute of Zoology; Institute for Stem Cell and Regeneration, Chinese Academy of Sciences, Beijing, China; ^3^Beijing Institute for Stem Cell and Regenerative Medicine, Beijing, China; ^4^University of Chinese Academy of Sciences, Beijing, China

**Keywords:** placenta, miRNA, exosome, pregnancy, preeclampsia

## Abstract

In placental mammals, reproductive success, and maternal-fetal health substantially depend on a well-being placenta, the interface between the fetus and the mother. Disorders in placental cells are tightly associated with adverse pregnancy outcomes including preeclampsia (PE), fetal growth restriction, etc. MicroRNAs (miRNAs) represent small non-coding RNAs that regulate post-transcriptional gene expression and are integral to a wide range of healthy or diseased cellular proceedings. Numerous miRNAs have been detected in human placenta and increasing evidence is revealing their important roles in regulating placental cell behaviors. Recent studies indicate that placenta-derived miRNAs can be released to the maternal circulation via encapsulating into the exosomes, and they potentially target various maternal cells to provide a hormone-like means of intercellular communication between the mother and the fetus. These placental exosome miRNAs are attracting more and more attention due to their differential expression in pregnant complications, which may provide novel biomarkers for prediction of the diseases. In this review, we briefly summarize the current knowledge and the perspectives of the placenta-derived miRNAs, especially the exosomal transfer of placental miRNAs and their pathophysiological relevance to PE. The possible exosomal-miRNA-targeted strategies for diagnosis, prognosis or therapy of PE are highlighted.

## Introduction

The placenta is a transient organ that plays a central role in maternal and fetal health during pregnancy (Anin et al., 2004). Serving as the interface between the fetal and maternal environments, human placenta performs many critical functions throughout the gestation, such as exchange of gases, nutrients and waste products between the mother and the growing fetus (Cross, 1998). Human placenta is also an endocrine gland that modulates maternal physiological and metabolic events and provides an immune-protective milieu in which the semi-allogenic fetus can develop (Regnault et al., 2002). Human placenta develops from the trophectoderm, the outer layer of the pre-implantation embryo. Highly proliferative, undifferentiated primitive cytotrophoblast (CTB) cells that are derived from the trophectoderm give rise to differentiated trophoblast cells, mainly including villous syncytiotrophoblast (STB), cytotrophoblast (CTB), and extravillous trophoblast (EVT; Ji et al., 2013). Defects in placental development, especially the dysregulation of trophoblast differentiation, are tightly associated with fetal loss and pregnant complications, such as preeclampsia (PE), and fetal growth restriction (FGR; Knofler et al., 2019).

MicroRNAs (miRNAs) are endogenous, small non-coding single-stranded RNAs, on average 22nt in length, which can regulate gene expression primarily through post-transcriptional repression or messenger RNA degradation in a sequence-specific manner (Bartel, 2009). Most miRNAs are transcribed as precursors (either pri-miRNA or pre-mRNA) before capping and polyadenylation, and their biogenesis requires several enzymes, including Drosha, DGCR8, Dicer, and Argonaute (Ago) 2 (Donker et al., 2007). In recent years, the remarkable roles of miRNAs in cellular proceedings under healthy or diseased conditions have been increasingly recognized (Aghdam et al., 2019; Mirzaei and Hamblin, 2020; Sadri Nahand et al., 2020; Davoodvandi et al., 2021; Razavi et al., 2021). In particular, several studies have shown that knocking out the key enzymes in the miRNA processing results in embryonic arrest or even embryonic death (Bernstein et al., 2003; Alisch et al., 2007; Suh et al., 2010), indicating the significance of miRNAs in the regulation of pregnant process.

Human placenta is a transient organ with fast development characteristics and transcriptome diversity. By far, over 1000 mature miRNAs are identified in human genomes (Friedlander et al., 2014), among which more than 600 miRNAs have been found in human placenta (Mouillet et al., 2015), and a series of differential miRNAs have been demonstrated in the placentas from complicated pregnancies including PE (Pineles et al., 2007; Ura et al., 2014; Xu et al., 2014). In addition, *in vitro* and *in vivo* studies have revealed the vital roles of these placenta-derived miRNAs in the regulation of trophoblast cell behaviors and the occurrence of PE (Xu et al., 2014; Awamleh and Han, 2020; Dong et al., 2020). In addition to the intracellular silencing functions, an attractive feature of the placenta-derived miRNAs is their capability of releasing to the maternal circulation via being encapsulated into the exosomes, and thus potentially targeting various maternal cells to provide a hormone-like means of intercellular communication between the mother and the fetus (Chen et al., 2012).

In this article, we briefly summarize the current knowledge of the placenta-derived miRNAs, especially the exosomal transfer of placental miRNAs and their pathophysiological relevance to PE. The miRNA-targeted promising strategies for the diagnosis, prognosis or therapy of PE are highlighted.

## Expression and Function of Placenta-Derived miRNAs During Pregnancy

Placental development is a complicated process during which various subtypes of cells dynamically differentiate and interact with each other throughout gestation (Ji et al., 2013). The expression timing and cellular localization of miRNAs may change along gestation, indicating their time-dependent, and/or cell-type-dependent working mechanisms in the placenta. This point has been well-reflected in many studies. For instance, the higher expression of placental miR-18a at early gestation, as well as its specific localization in invasive EVTs are in consistence with its functions to regulate trophoblast cell invasion through targeting TGF-β/Smad2 signaling (Xu et al., 2020). The hypoxia-induced miRNA, miR-210, is transcribed in various subtypes of placental trophoblasts at early gestation in human beings and mice. It participates in modulating trophoblast cell proliferation, invasion, apoptosis, syncytialization, and angiogenesis by targeting various genes (Anton et al., 2013; Wang H. et al., 2020). Deficiency in miR-210 leads to failure in the response of the placenta to maternal hypoxia, especially at early fetal growth stage (Bian et al., 2020).

To date, emerging evidence has demonstrated the significance of miRNAs as regulators of various cell behaviors in human placenta. For instance, let-7a, miR-377, miR-675, miR-145, and miR-518b, etc., are involved in the regulation of trophoblast cell proliferation (Forbes et al., 2012; Gao et al., 2012; Doridot et al., 2013; Liu et al., 2018), miR-34a, miR-29b, miR-376c, miR-195, miR-210, and many others have roles in modulating trophoblast cell differentiation toward the invasive pathway (Pang et al., 2010; Fu et al., 2013; Li et al., 2013; Luo et al., 2014; Wu et al., 2016). The placental steroidogenesis can be regulated by miR-210, miR-518c, and miR-22 (Ishibashi et al., 2012; Shao et al., 2017), and the mitochondrial respiration activities and apoptosis of placental cells are associated with miR-210 and miR-195 (Wang et al., 2018; Anton et al., 2019). However, these functional outcomes are vastly based on *in vitro* studies using various cell models, and the relevant *in vivo* evidence using genetically manipulated mouse models has been largely lacking.

Among the placenta-derived miRNAs, there exists a placenta-specific miRNA cluster termed the chromosome 19 miRNA cluster (C19MC). The C19MC is the largest cluster of miRNAs in the human genome, and contains 46 highly homologous miRNA genes within a ∼100 kb genomic region (Bortolin-Cavaille et al., 2009). The miRNAs in this cluster are predominantly expressed in the primate placenta and some fetal tissues as well as various tumor cells (Bentwich et al., 2005; Zhang et al., 2008; Setty et al., 2020). During pregnancy, they are highly expressed in placental trophoblasts, and released into maternal circulation which are eliminated after delivery (Luo et al., 2009; Donker et al., 2012). Although the full repertoire of the biological actions of C19MCs remains to be established, a recent study by Mouillet et al. (2020) have proved the roles of one of the C19MC members, miR-519d-3p, in promoting trophoblast cell proliferation and decreasing cell migration abilities. In addition, C19MC miRNAs are detected in embryonic stem (ES) cells, and their expression drops considerably when ES cells begin to differentiate, indicating their roles in the maintenance of the undifferentiated status (Stadler et al., 2010). Several members of C19MC miRNAs, such as miR-519, miR-517a, and miR-517c, also exhibit tumor-suppressive activity via triggering cell senescence (Marasa et al., 2010) or inhibiting cell proliferation (Liu et al., 2013).

## Secretion and Function of Placental miRNAs in Exosomes

Exosomes are small extracellular vesicles of endocytic origin (van der Pol et al., 2016). They can be released by many cells and are found in body fluids, including peripheral blood, lymph, and milk, etc. (Akers et al., 2013). The significance of exosomes in the progression, diagnosis and treatment of various diseases have been suggested (Asgarpour et al., 2020; Ghaemmaghami et al., 2020; Hashemian et al., 2020; Nahand et al., 2020). Interestingly, during pregnancy, the number of exosomes in maternal plasma appears to be significantly increased from the first trimester (Sarker et al., 2014), and reaches a maximum level at term (Jin and Menon, 2018). It is estimated that the concentration of exosomes in maternal peripheral blood is 20-fold higher than non-pregnant control (Sabapatha et al., 2006), and returns to non-pregnant levels within 48 h of delivery (Salomon et al., 2014). In pregnant complication such as PE, the level of maternal circulating exosome is progressively higher than normal pregnant controls (Chiarello et al., 2018).

**FIGURE 1:**
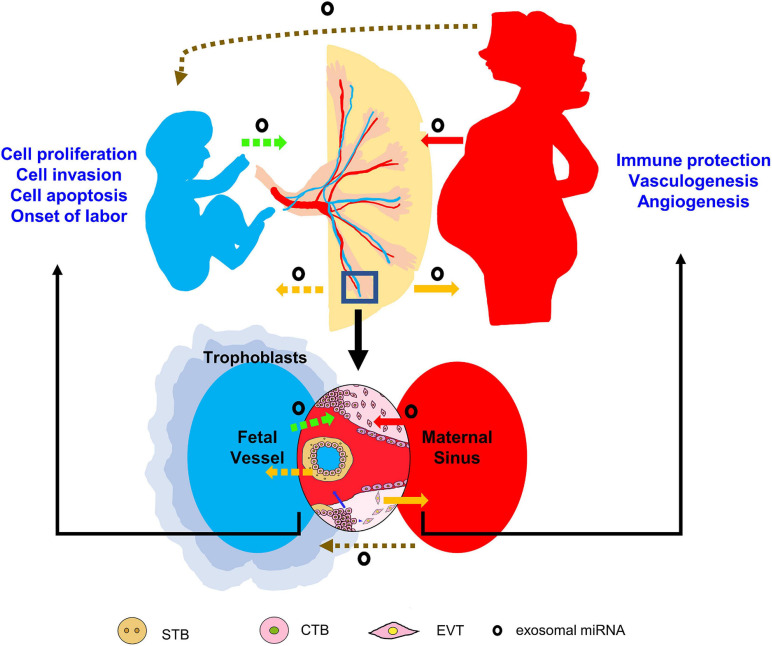
Potential trafficking routes and working mechanisms of exosomal miRNAs during pregnancy. Placenta-derived exosomal miRNAs may be delivered to maternal or fetal circulation, and affect various cell events in targeting organs. The mother or fetus-derived exosomal miRNAs may also traffic to the placenta to regulate placental development. Note the dashed lines indicate the uncertainty of evidence by far, the blue arrow indicates endocrine manner and the blue arrow head indicates paracrine manner.

Exosomes contain multifaceted cargoes, including proteins, lipids, DNAs, mRNAs, miRNAs, LncRNAs, tRNA, and tRNA associated fragments (Sarker et al., 2014; Jeppesen et al., 2019). The selective sorting of miRNAs into exosomes is attributed to the help of specific RNA-binding proteins, such as hnRNPA2B1 and Ago-2. Other membrane proteins including Caveolin-1 and Neural Sphingomyelinase 2 are also involved in this process (Groot and Lee, 2020). The observations by Valadi et al. (2007) first demonstrated the mechanisms of genetic exchange between different cells by the exosome transfer of miRNAs. Later on, Luo et al. (2009) indicated the extracellular release of placental miRNAs via exosomes into maternal blood. By far, accumulating studies have identified many exosome-packaged placental miRNAs and their release into extracellular compartments and maternal blood (Kambe et al., 2014; Ouyang et al., 2014; Mitchell et al., 2015; Chang et al., 2017; Chiarello et al., 2018; Zhao et al., 2018; Czernek and Duchler, 2020; Li et al., 2020; Yadava et al., 2020; Yang et al., 2020; Wang Y. et al., 2020). The placental exosomal miRNAs may target other cells at the feto-maternal interface in paracrine manner (Takahashi et al., 2017; Wang Y. et al., 2020), or transfer to maternal recipient cells and play endocrine functions (Kambe et al., 2014; Zhao et al., 2018; Ma et al., 2020). What’s more, bidirectional trafficking of exosomal miRNAs between the placenta and the fetal compartment has been suggested (Chang et al., 2017; Shen et al., 2018; Yang et al., 2019; Yadava et al., 2020; Wang D. et al., 2020). We summarize the recognition of the placental exosomal miRNAs in Figure 1, and example some representative studies as below:

1) The placenta-derived exosomal miRNAs transfer to the maternal circulation and modulate maternal immune cells to protect the fetus from the maternal immune attacks. *In vivo* and *in vitro* studies have demonstrated the dramatical repression of PRKG1 expression in maternal NK cells by exosomal miR-517-3p (Kambe et al., 2014), and the downregulation of IL-24 and thus the suppression in the proliferative capacity and anti-inflammatory effect of macrophage by exosomal miR-203a-3p (Ma et al., 2020). In cattle, the placental exosome-derived bta-miR-499 inhibits the activation of NF-κB via Lin288/let-7 axis, thereby attenuates the inflammatory responses and forms an immune-tolerant microenvironment in the uterus. Inhibition of miR-499 lead to inflammatory deregulation and increased risk of pregnancy failure (Zhao et al., 2018).
2) Trophoblast cell behaviors can be regulated by exosomal miRNAs derived from the neighboring or distant placental cells. Exosomal miR-520c-3p of villous CTB origin can inhibit cell invasiveness via downregulating CD44 expression levels in targeted EVT cells (Takahashi et al., 2017). *In vivo* and *in vitro* results indicate the roles of placental exosomal miR-15a-5p in suppressing trophoblast cell proliferation, invasion, and apoptosis through downregulating CDK1 expression and hampering PI3K/AKT signaling, which is closely associated with PE progression (Wang Y. et al., 2020).
3) The placenta-derived exosomal miRNAs may regulate fetal vasculogenesis and angiogenesis. A study from Shen et al. (2018) showed the downregulation of eNOS expression in human umbilical vein endothelial cells by exosomal miR-155 of placenta origin, indicating the potential delivery of placental miRNAs to the fetal part. However, *ex vivo* or *in vivo* evidence that supports the transfer of placental exosomes from the placenta to the fetus remains largely lacking.
4) A potential mode of exosomal miRNAs-mediated fetus-to-placenta signaling has been suggested. For instance, miR-133b in human umbilical cord mesenchymal stem cell (MSC)-derived exosomes boosts trophoblast cell proliferation, migration, and invasion via targeting SGK1 gene (Wang D. et al., 2020). Exosomal miR-146a-5p and miR-548e-5p derived from amniotic fluid-MSCs exert anti-inflammatory effects on human trophoblast cells, and their dysregulation are associated with the occurrence of preterm birth (Yang et al., 2019). Umbilical artery-derived miR-15b-5p can be delivered to the placenta, and can repress the expression levels of Aplein and cytokines including IL-1, IL-6, IL-8, and TNF-α, and thus is believed to play roles in the onset of labor (Yadava et al., 2020). In addition to target trophoblast cells, the maternal and umbilical cord blood-derived exosomes can effectively influence endothelial cells, which is closely associated with the encapsulation of miRNAs into the exosomes (Jia et al., 2018). In pigs, miR-150 in umbilical cord blood-derived exosomes exhibits a pro-angiogenic effect by stimulating the proliferation and migration of endothelial cells. A reduced expression of this exosomal miRNA leads to intrauterine growth restriction of the fetus (Luo et al., 2018). However, more evidence from appropriate *in vivo* models are needed to clarify the working mechanisms of exosome transfer from the fetus to the placenta.

## Clinical Implications of the Placenta-Derived miRNAs for PE

### Exploring the Pathogenesis of PE Using Placenta-Derived miRNAs

Preeclampsia has long been the leading cause of maternal and fetal morbidity and mortality, affecting approximately 2–7% of pregnancies. It is defined as the sudden onset of hypertension after the 20th week of gestation in pregnant women who had no preexisting hypertension, accompanied by significant proteinuria or multi-system symptoms, such as pulmonary oedema, seizures, or oliguria (Sibai, 2003). A well-accepted theory is that defects in placenta development, especially the dysregulation of trophoblastic behaviors, are predominant causes of the disease.

Great efforts have been put to identify genes or signaling pathways that are associated with the deregulation of trophoblast differentiation and the development of PE (Ji et al., 2013; Staff, 2019). A great number of differential miRNAs in PE placentas have been screened, and a series of *in vivo* and *in vitro* results have demonstrated the participation of these aberrantly expressed miRNAs in PE-associated placental defects (Pang et al., 2010; Ji et al., 2013; Anton et al., 2019; Xu et al., 2020).

For PE, a big challenge is the discrepancy between the key pathophysiological changes that are initiated well before the 20th week of gestation and the clinical symptoms that are not manifested until after that. Therefore, a critical thought is whether these differential placental miRNAs really contribute to the pathological change of PE or they are just the consequences of the disorder at late gestation (Baker and Delles, 2013). As stated above, placental miRNAs can be released to maternal circulation during pregnancy in pregnant women (Luo et al., 2009). Identification of the differential miRNAs in maternal blood at early-to-mid gestation in PE patients may provide valuable hints of the pathophysiological placental factors (Gunel et al., 2011). Our previous results have revealed several miRNAs (including miR-376c, miR-18a, miR-19b1, miR-92a1, miR-210, and miR-195) that exhibit significantly aberrant concentrations in the plasma of PE patients from gestational weeks 15–19 to term. These miRNAs in the placenta potentially contribute to compromised cell differentiation and functional homeostasis (Fu et al., 2013; Xu et al., 2014; Wang et al., 2018). However, results of genetic manipulation of these small RNAs in mice are lacking. Knocking down of miR-210 leads to relatively weak influence on fetal development (Krawczynski et al., 2016; Bian et al., 2020). This may reflect the fine-tune features of miRNAs to maintain cellular homeostasis, and also indicate the complicated compensatory routes of various placental miRNAs as well.

### Circulating miRNAs Are Promising Biomarkers for the Prediction of PE

Circulating miRNAs can be encapsulated into extracellular vesicles including exosomes or bound to stabilizing proteins (mainly Ago proteins; Arroyo et al., 2011). Plasma miRNAs (including the vesicular form and the non-vesicular form) are relatively stable, being not affected by experimental conditions such as incubation temperature, pH or even RNase A treatment (Chen et al., 2008; Mitchell et al., 2008; Arroyo et al., 2011). The vesicle-packaged miRNAs are more resistant to degradation. Although the exosomal miRNAs constitute only a fraction of the whole plasma miRNA population, they exhibit unique changing pattern PE patients (Biro et al., 2019; Li et al., 2020). Since exosomal miR-885-5p is suggested as the potential predictive marker for PE (Sandrim et al., 2016), increasing attention has been put into this emerging field. The unique characteristics of exosomal miRNAs make them rather promising as non-invasive biomarkers for diagnosing or monitoring the development of PE. We summarize the relevant progress in Table 1. Notably, studies from Motawi et al. (2018) indicate significant increase in miR-136, miR-494 and miR-495 in circulating exosomes from PE patients before the 20th week of gestation, and receiver operating characteristic curve analysis reveals promisingly high sensitivity and specificity of these miRNAs to predict PE before the onset of clinical manifestations.

**TABLE 1:** A brief summary of the differential exosomal miRNAs in the plasma from PE patients.

**Exosomal miRNAs**	**Gestational weeks**	**Sample size**	**Method**	**Changes in PE plasma**	**Diagnostic capability**	**References**
miR-885-5p	11–14 weeks	Selection cohort: PE (*n* = 19) and control (*n* = 14); Validation cohort: PE (*n* = 8) and control (*n* = 8)	NGS and qRT-PCR	↑	–	Sandrim et al., 2016
miR-136	<20-week gestation	PE (*n* = 20) and control (*n* = 23)	qRT-PCR	↑	AUC = 1.00, Sen = 95.00%, Spe = 100.00%	Motawi et al., 2018
miR-494	<20-week gestation	PE (*n* = 20) and control (*n* = 23)	qRT-PCR	↑	AUC = 0.87, Sen = 86.00%, Spe = 95.00%	Motawi et al., 2018
miR-495	<20-week gestation	PE (*n* = 20) and control (*n* = 23)	qRT-PCR	↑	AUC = 0.94, Sen = 90.00%, Spe = 83.00%	Motawi et al., 2018
miR-153-3p	Term	PE (*n* = 20) and control (*n* = 23)	Taqman qPCR	↑	–	Li et al., 2020
miR-222-3p	Term	PE (*n* = 20) and control (*n* = 23)	Taqman qPCR	↓	–	Li et al., 2020
miR-224-5p	Term	PE (*n* = 20) and control (*n* = 23)	Taqman qPCR	↓	–	Li et al., 2020
miR-325	Term	PE (*n* = 20) and control (*n* = 23)	Taqman qPCR	↑	–	Li et al., 2020
	–	PE (*n* = 20) and control (*n* = 23)	qRT-PCR	↑	–	Wang Y. et al., 2020
miR-342-3p	Term	PE (*n* = 20) and control (*n* = 23)	Taqman qPCR	↑	–	Li et al., 2020
miR-532-5p	Term	PE (*n* = 20) and control (*n* = 23)	Taqman qPCR	↓	–	Li et al., 2020
miR-653-5p	Term	PE (*n* = 20) and control (*n* = 23)	Taqman qPCR	↑	–	Li et al., 2020
miR-203a-3p	–	PE (*n* = 36) and control (*n* = 30)	qRT-PCR	↓	–	Ma et al., 2020
miR-134	<13-week gestation	PE (*n* = 4) and control (*n* = 5)	miRNA array	↑	–	Devor et al., 2020
miR-196b	26–40 weeks	PE (*n* = 4) and control (*n* = 5)	miRNA array	↓	–	Devor et al., 2020
miR-302c	26–40 weeks	PE (*n* = 4) and control (*n* = 5)	miRNA array	↑	–	Devor et al., 2020
miR-346	26–40 weeks	PE (*n* = 4) and control (*n* = 5)	miRNA array	↑	–	Devor et al., 2020
miR-376c	<13-week gestation	PE (*n* = 4) and control (*n* = 5)	miRNA array	↑	–	Devor et al., 2020
miR-486-3p	<13-week gestation	PE (*n* = 4) and control (*n* = 5)	miRNA array	↑	–	Devor et al., 2020
miR-590-5p	<13-week gestation	PE (*n* = 4) and control (*n* = 5)	miRNA array	↑	–	Devor et al., 2020
miR-618	26–40 weeks	PE (*n* = 4) and control (*n* = 5)	miRNA array	↑	–	Devor et al., 2020
miR-155	Term	PE (*n* = 10) and control (*n* = 10)	qRT-PCR	↑	–	Shen et al., 2018
miR-486-1-5p	The whole gestation	PE (*n* = 15) and control (*n* = 32)	NGS	↑	–	Salomon et al., 2017
	–	PE (*n* = 20) and control (*n* = 10)	qRT-PCR	↑	–	Wang Y. et al., 2020
miR-486-2-5p	The whole gestation	PE (*n* = 15) and control (*n* = 32)	NGS	↑	–	Salomon et al., 2017
	–	PE (*n* = 20) and control (*n* = 10)	qRT-PCR	↑	–	Wang Y. et al., 2020
miR-125a-5p	After the diagnosis of PE	PE (*n* = 18) and control (*n* = 20)	qRT-PCR	↑	–	Xueya et al., 2020
miR-423-5p	–	PE (*n* = 20) and control (*n* = 10)	qRT-PCR	↑	–	Wang Y. et al., 2020
miR-451a	–	PE (*n* = 20) and control (*n* = 10)	qRT-PCR	↑	–	Wang Y. et al., 2020
miR-15a-5p	–	PE (*n* = 20) and control (*n* = 10)	qRT-PCR	↑	–	Wang Y. et al., 2020
miR-92a-1-3p	–	PE (*n* = 20) and control (*n* = 10)	qRT-PCR	↑	–	Wang Y. et al., 2020
miR-92a-2-3p	–	PE (*n* = 20) and control (*n* = 10)	qRT-PCR	↑	–	Wang Y. et al., 2020
miR-103a-1-3p	–	PE (*n* = 20) and control (*n* = 10)	qRT-PCR	↑	–	Wang Y. et al., 2020
miR-103a-2-3p	–	PE (*n* = 20) and control (*n* = 10)	qRT-PCR	↑	–	Wang Y. et al., 2020
miR-126-3p	–	PE (*n* = 20) and control (*n* = 10)	qRT-PCR	↑	–	Wang Y. et al., 2020
miR-520a-5p	10–13 weeks	PE (*n* = 43) and control (*n* = 50)	qRT-PCR	↓	AUC = 0.63, Sen = 60.47%, Spe = 70.00%	Hromadnikova et al., 2019
miR-517-5p	10–13 weeks	PE (*n* = 43) and control (*n* = 50)	qRT-PCR	↓	AUC = 0.699, Sen = 60.47%, Spe = 84.00%	Hromadnikova et al., 2019
miR-525-5p	10–13 weeks	PE (*n* = 43) and control (*n* = 50)	qRT-PCR	↓	AUC = 0.698, Sen = 51.16%, Spe = 84.00%	Hromadnikova et al., 2019
miR-210	PE (24–39 weeks) and control (30–39 weeks)	PE (*n* = 19) and control (*n* = 34)	qRT-PCR	↑	–	Biro et al., 2017
	PE (31.00 ± 5.07 weeks) and control (36.13 ± 3.00 weeks)	PE (*n* = 8) and control (*n* = 8)	qRT-PCR	No significant change	–	Biro et al., 2019

*PE, preelampsia; NGS, next generation sequencing; qRT-PCR, reverse transcription-real-time quantitative polymerase chain reaction; Taqman-qPCR, Taqman quantitative polymerase chain reaction; AUC, area under curve; Sen, sensitivity; and Spe, specificity.*

It has to be noticed that the results from various studies may show different changing patterns of the exosomal miRNAs in PE patients (Biro et al., 2017, 2019). The variations may attribute to the differences in sample size, the gestational week at sampling, or the statistical method. Importantly, the studies using large-scale plasma samples in detailed time points during gestation with normalized statistical methods are needed to achieve reproducible results and confirm the clinical sensitivity and specificity of exosomal miRNAs as the promising biomarkers for PE.

### Exosomal miRNAs Are Potentially Useful Tools for the RNA-Based Therapies for PE

In recent years, RNA-based medicine is receiving growing attention for its diverse roles and promising therapeutic capacity (Chow et al., 2020). Interestingly, the exosomes can be engineered to load with miRNAs of interest and delivered to the recipient cells and/or organs, reinforcing the possibility to tailor exosomes as gene-delivery vehicles (Thomou et al., 2017). One technology-barrier is difficulties in introducing anti-miR into exosomes and delivering anti-miR to exosome-recipient cells after intravenous administration. Recently, Yamayoshi (2020) have constructed a novel drug delivery system using anti-exosome antibody-oligonucleotide conjugates to functionally inhibit circulating miRNAs, which sheds light on developing strategy for PE treatment.

## Conclusion and Perspectives

The discovery of placenta-derived miRNAs and their multiple roles in maintaining healthy pregnancy undoubtedly represent one of the most exciting progresses in recent years. In addition to the canonical intracellular silencing functions, placenta-derived miRNAs can also be released to the maternal or fetal circulation via encapsulating into the exosomes, and therefore potentially target various recipient cells to provide a non-hormonal means of intercellular communication between the mother and the fetus. Furthermore, unique exosomal miRNA profiling is potential diagnostic or predictive and prognostic tool for pregnant complications such as PE, and provides novel treatment targets for the disease.

There exist several interesting topics that require further investigations. First, studies regarding exosomal miRNA in pregnant women have been predominantly focusing on the total exosomal miRNAs, while seldom identifying their diverse origins. A recent report reveals that the origin of exosomes determines its target cells and the transfer activity (Sancho-Albero et al., 2019), indicating the importance of further clarifying whether the circulating exosomal miRNAs in the pregnant women are derived from the placenta, the fetus or various maternal organs. This may greatly deepen our understanding of the mechanisms underlying the complicated fetal-maternal interactions during gestation. To follow this concern, the precise trafficking routes and the specific targeting cells or organs of the placental exosomal miRNAs are yet to be clarified. Proper *in vivo* and *ex vivo* models should be constructed to address this point, which is indispensable for developing exosomal miRNA-based therapeutic strategies for pregnant complications such as PE. Finally, the convenient and controllable detection of exosomal miRNA remains challenging, because the adequately simple and robust assay platforms are lacking. A recent wok by Xia et al. (2021) has developed a colorimetric strategy to detect exosomal miR-21 by switching the visible-light-induced oxidase mimic activity of acridone derivate. This may provide a feasible tool for the application in exosomal miRNAs-based diagnosis of PE.

## Author Contributions

PX and YM drafted the manuscript. HW participated in reference mining. Y-LW designed and supervised the study, and revised the manuscript. All authors contributed to the article and approved the submitted version.

## Conflict of Interest

The authors declare that the research was conducted in the absence of any commercial or financial relationships that could be construed as a potential conflict of interest.
